# Maintaining Resilience and Well-Being in the Era of Climate Change: Protocol of an Acceptability and Feasibility Pilot of the Bee Well Program for Treating Eco-Anxiety in Rural Children Exposed to Natural Hazards

**DOI:** 10.2196/69005

**Published:** 2025-07-18

**Authors:** Suzanne M Cosh, Warren Bartik, Rosie Ryan, Amanda Jefferys, Kaii Fallander, Phillip J Tully, Amy D Lykins

**Affiliations:** 1 School of Psychology University of New England Armidale Australia; 2 School of Psychology The University of Adelaide Adelaide Australia; 3 School of Psychology Deakin University Geelong Australia; 4 School of Medicine The University of Adelaide Adelaide Australia

**Keywords:** climate change anxiety, coping, climate emotions, eco-distress, eco-grief, treatment, youth, adolescents, young people, rural mental health

## Abstract

**Background:**

The effects of climate change on mental health are becoming widely recognized. Mental health can be impacted through direct and indirect exposure to natural hazards, as well as through the overarching awareness of climate change and the resultant environmental decline—the latter is termed eco-anxiety. Exposure to natural hazards also increases eco-anxiety, further compounding mental health impacts. Young people are especially vulnerable to the mental health impacts of climate change and have higher rates of eco-anxiety than other age groups. Those in rural areas are also more likely to be impacted by natural hazards, further underscoring the need to support this population. To date, there remains scant evidence regarding how to support young people with eco-anxiety, and few interventions, especially for children, have been evaluated. There is a need for further research to inform treatment for young people for climate change–related distress.

**Objective:**

This study pilots a novel group-based mental health and resilience intervention in relation to eco-anxiety. Specifically, this project aims to explore the acceptability, feasibility, and clinical utility of a group-based eco-anxiety intervention.

**Methods:**

The project is an exploratory pilot assessing the acceptability, feasibility, and clinical utility of a group-based intervention using a pre-post design with a single group. A minimum sample of 12 children aged 10-14 years located in a rural area and with exposure to at least one natural hazard will be enrolled in this study. In order to assess clinical utility, changes from preintervention to postintervention in distress, resilience, and climate emotions will be assessed. To do so, children will complete measures of psychological distress (subjective units of distress, Depression Anxiety Stress Scale-21–youth version), climate emotions, and resilience (Resilience Scale for Children-10) before and after the intervention. Acceptability will be assessed post intervention through a series of Likert scale and open-ended questions. Feasibility will be assessed through enrollment and the proportion of participants completing the full intervention. Eligible children will take part in a novel 5-module group-based intervention designed to build resilience, promote nature connectedness, build social support, and foster meaning-focused coping.

**Results:**

This study has received ethics board approval by the University of New England’s Human Research Ethics Committee (HE23-080). This study will be conducted from late 2024 to 2025. As of March 2025, 28 children have been enrolled in the study.

**Conclusions:**

Rural children and young people are an especially vulnerable population for the mental health impacts of climate change. To date, the evidence base for interventions for treating eco-anxiety remains sparse, especially for young people and children who typically have higher rates of eco-anxiety than older age groups. This study will provide preliminary evidence of a group-based treatment for children and adolescents experiencing eco-anxiety that can inform practitioners.

**Trial Registration:**

Australia and New Zealand Clinical Trials Registry ACTRN12624001287527; https://www.anzctr.org.au/Trial/Registration/TrialReview.aspx?id=388545

**International Registered Report Identifier (IRRID):**

PRR1-10.2196/69005

## Introduction

### Climate Change and Mental Health

Climate change is an ecological crisis that poses a significant threat to human health [1]. It has been widely reported that climate change can negatively impact mental health [2,3], both through direct and indirect exposure to natural hazards, as well as through the overarching awareness of climate change and the associated environmental decline, known as eco-anxiety [4]. The detrimental impacts of exposure to natural hazards such as bushfires and drought on mental health and well-being have been widely reported and can be enduring [5-7]. An emerging body of research has also routinely highlighted the deleterious impact of eco-anxiety on a range of mental health outcomes [8,9]. As climate change continues to accelerate the intensity, frequency, and severity of climate-related natural hazards [10], there is an increased likelihood of exposure and repeated exposures to these events. Direct exposure to natural hazards also increases climate change distress and concern [11], compounding exposure-related distress and leaving exposed individuals especially vulnerable to poor mental health outcomes.

### Climate Change and Young People

Although much of the existing literature has explored the mental health outcomes of adults, children and young people are considered to be a priority at-risk group in relation to climate change [12,13]. Children are likely to be the most affected by climate change [14,15] and from early and developmentally significant life stages [16] while having contributed minimally to the climate crisis. Young people around the world report high levels of climate change concern [17], with higher levels of eco-anxiety observed in young people than in older age groups [18-21]. These high rates of eco-anxiety are also related to distress and poor mental health in children [22,23].

Young people are also especially vulnerable to direct and indirect exposure to natural hazards [24], in part due to exposure to parental stress [25,26] and erosion of normal support systems [27] at an age where coping skills are still emerging [28]. Recent evidence highlights high levels of depression, stress, anxiety, substance misuse, adjustment disorder symptoms, and low resilience in young people following the Black Summer 2019-2020 bushfires in Australia, especially for those who were directly exposed [11]. Children and young people residing in rural areas are further at increased susceptibility to poor mental health in relation to climate change due to (1) already higher rates of psychological distress compared with their metropolitan counterparts [29], (2) more likely exposure to adverse climate change–related hazard events, and (3) limited mental health service access in rural areas [30]. Despite the body of evidence pointing to the impacts of climate change on mental health, evidence to guide practice remains scant, with few interventions for eco-anxiety currently evaluated [3,31].

### Climate Change and Resilience

Psychological resilience is a key protective factor for mental health and is understood as a process of adaptation to a stressor [32]. It has been argued that building resilience may be a key way to protect against impacts of climate change on mental health [33-35]. Climate change resilience—the ability to prepare for, adapt, and respond to climate-related hazards such as extreme weather events [36]—has most commonly focused on physical resilience, with less emphasis to date on how to build or foster psychological climate-resilience (ie, the ability to psychologically adapt to climate-related stressors).

Although psychological resilience has been widely studied, how to best build resilience and how resilience intersects with broader social factors and challenges such as climate change remains less well established [32]. Concomitantly, research examining how to build psychological climate-resilience remains in the nascent stages. This study thus crucially builds the literature base around developing psychological climate resilience in children in order to protect mental health.

### Interventions

To date, evidence for interventions to support climate change–related distress remain scant, and interventions proposed to date lack empirical assessment. Suggestions from the limited literature point to fostering hope and optimism [37] and empowering young people to feel a sense of efficacy to support coping—such as through taking climate action—as potentially beneficial [22,24]. Meaning-focused coping in relation to climate change has been shown to predict better mental health outcomes in young Swedes [38], suggesting the potential value of fostering this specific coping style. However, allowing children to explore and express negative emotional states is also critical [39]. Additionally, the use of ecotherapy, enhancing connection with nature [31,40], and mindfulness, alongside psychoeducation [33] have also been proposed. Group-based approaches may also be well placed to treat eco-anxiety, allowing for social support [31]. Given that the mental health care sector will struggle to cope with the impacts of climate change [33,41,42], group-based approaches may also be more feasible as a treatment modality. However, little is known regarding the effectiveness of any eco-anxiety–informed interventions, with minimal evaluation of any interventions currently undertaken [31].

### Goal of This Study

This study pilots a novel group-based mental health and resilience intervention in relation to eco-anxiety for rurally located children who have been impacted by natural hazards. Specifically, the objective of this project is to explore the acceptability, feasibility, and clinical utility of a group-based eco-anxiety intervention.

## Methods

### Design

This project is an exploratory trial to assess the acceptability, feasibility, and clinical utility of a group-based intervention using a pre-post design with a single group (trial registration ACTRN12624001287527). This protocol is presented in line with SPIRIT (Standard Protocol Items: Recommendations for Interventional Trials) reporting guidelines [43].

### Participants

#### Sample Size

Being an intervention pilot precludes definitive sample size calculation [44]. However, it has been argued that, for clinical intervention pilots, a minimum of 10 samples in the intervention group is required [45]. In order to allow for up to 20% attrition and ensure sufficient data for preliminary efficacy testing, a minimum sample of 12 is anticipated, which is sufficient to provide acceptability and feasibility data and preliminary data regarding clinical utility [44].

#### Setting

This pilot study is conducted in a rural region of northern New South Wales, Australia, with a population of approximately 80,000 people. This area is largely agricultural-dependent. The study is conducted in a community setting.

#### Eligibility

##### Inclusion Criteria

Persons aged 10-14 years, of any gender, living in a rural area (defined as areas with a Modified Monash Model 3 or higher, representing large rural towns through to remote communities) with self-reported exposure to one or more natural hazards (eg, fire, flood, tornado, drought) are included in this study. Given that the impacts of hazard exposure can be sustained well past the initial exposure [5,6], there are no time limits on exposure to the hazards. There are no restrictions on participants currently or previously having accessed other mental health interventions.

##### Exclusion Criteria

Persons outside of the age range, not residing in a rural area, or without exposure to a natural hazard are excluded from this study. Individuals who fail comprehension checks as ascertained by clinicians during provision of child assent or who have acute suicidal risk or a cognitive impairment/disability as reported by parents are also excluded.

#### Recruitment

Recruitment will occur through rural mental health and health care services, as well as through community groups and organizations and school-based well-being officers and psychologists. In particular, recruitment includes placing flyers and sharing project information with local Headspace and health centers, the University psychology clinic (which receives referrals for young people), and local youth organizations. Additionally, the project was presented to school psychologists and well-being officers to refer children to the project. Recruitment commenced in late 2024, with 28 children currently enrolled in the study.

### Intervention

#### Development

Taking the best available evidence for treating eco-anxiety in young people and extensive consumer consultation and co-design, a group-based eco-anxiety intervention was developed. An expert and consumer advisory group consisting of a range of professionals, including teachers, psychologists, school well-being officers, environmental psychology researchers, and young end users provided co-design input into the development and refinement of the intervention manuals [46,47] and workbooks [48,49]. The Bee Well intervention has been developed both for teenagers (15-18 years) and children/adolescents (10-14 years); this protocol relates only to the child/adolescent version of the intervention.

#### Intervention Overview

The Bee Well intervention is informed by the resilience-building empirical evidence, alongside emerging evidence surrounding coping with climate change [37,38], which also points to the value of ecotherapy, nature connectedness, and nature immersion [40]. The intervention is designed to be run in a group setting in order to normalize climate change–related emotions and foster social support [31]. The intervention takes place predominantly outdoors to enable nature exposure and connection and consists of guided discussion and activities that focus on providing psychoeducation on emotional awareness and climate emotions, developing adaptive coping strategies for coping with climate change distress and hazards, and building resilience (see Table 1). The intervention also includes a nature restoration–based activity, occurring alongside discussions around meaning-making and coping, in order to foster efficacy and engage in shared action. Group members have their own individual workbooks and complete a range of individual and group activities, in addition to the nature restoration task. The intervention consists of 5 modules, with each module lasting approximately 60 minutes and will be co-delivered by a team of provisional and clinical psychologists with an interest in child and adolescent mental health, rural health, and/or eco-anxiety.

**Table 1:** Intervention modules.

Module	Description	Purpose
Module 1: Finding Common Ground	In this module, individuals explore and share their own eco-emotions, including grief, worry, and hope, through 2 interactive group activities (exploring eco-emotions and exploring mixed feelings).	Psychoeducation regarding emotions; build emotional awareness, recognition of shared experiences, and enable group processing
Module 2: Taking Action Together	In this module, the group commences a shared nature restoration activity—flora-based or fauna-based—such as building bee hives. While engaging in the activity, the group also undertakes guided discussions regarding “taking action” and exploring their own relationships with nature.	Taking action together through engaging in nature restoration as a form of coping and building group-efficacy and a means to support meaning making and fostering peer connection
Module 3: Coping	In this module, the group is introduced to coping skills through activities and group discussions. Group members undertake a mindfulness nature meditation/journaling task as a means of connecting with nature and rewilding.	Build adaptive coping skills, including promoting nature connectedness as a means of coping, and fostering resilience
Module 4: Cultivating Resilience	In this module, the group completes the nature restoration activity. Further discussions focus on fostering resilience and developing individual coping and resilience plans.	Build sense of community, group-efficacy, and self-efficacy; build resilience
Module 5: Bringing It Together	In this module, the group reflects on the nature restoration activity and discusses how this project will restore nature into the future. This module also includes a final reflection on the learnings and outlines avenues for ongoing or future support.	Reflection on the intervention and learnings

The Bee Well intervention resources can be found in the publicly available manual and workbook [47,49]. The intervention delivery is provided by trained psychologists who are able to manage referral or escalation, as needed, should any distress arise from or during participation. Information on referral sources is also provided to parents as part of the information sheet.

### Outcomes

Data will be collected before and after the intervention. The primary assessed outcomes will include changes in the psychological symptoms, resilience, and subjective ratings of distress, with secondary outcomes examining change in climate emotions, from preintervention to postintervention. Acceptability will also be assessed post intervention. Data will be collected by trained independent data collectors.

#### Psychological Distress

Psychological distress will be assessed using the Depression Anxiety Stress Scale (DASS)-youth version [50], which is a child and adolescent adaptation of the well-validated and widely used DASS-21 [51]. The measure consists of 21 items, with 7 items assessing each of the 3 conditions: depression, anxiety, and stress. Participants are asked to specify the frequency on a 4-point Likert scale (never to almost always), with which they have experienced symptoms. The DASS-youth version has replicated the original factor structure of the DASS-21 and has been validated for use in children aged 7-18 years [50].

#### Resilience

Resilience will be assessed using the Resilience Scale for Children [52]. This 10-item measure assesses individual resilience capacity in relation to 5 domains: sense of purpose and meaning, authenticity, equanimity, self-reliance, and perseverance. Respondents are asked to respond to a series of statements (eg, I think I’m okay just the way I am right now; when I get upset, I know how to calm down), indicating how well each statement described them. Items are scored on a 4-point Likert scale (1-4) from “not at all like me” to “a lot like me,” with higher scores suggesting greater resilience. The Resilience Scale for Children is validated for use in children as young as 7 years [52].

#### Climate Change Emotions

Two items will be used to assess distress in relation to climate change. The first asks participants to respond to the question “are you worried about climate change?” on a 5-point Likert scale ranging from “not worried” to “extremely worried.” The second item provides respondents with a list of 9 emotions (sad, helpless, worried, scared, hopeful, mad, hurt, powerless, and not bothered) and asks them to indicate if they feel each of these in relation to climate change (yes/no/prefer not to say). These items are adapted from Hickman et al [17]. The original items were designed for adolescents aged 16 years and older. Therefore, the wording of these items has been slightly modified to better suit the younger age range by psychologists with experience working with children and adolescents.

#### Subjective Units of Distress

This is a widely used short tool that asks respondent to rate how they are currently feeling. Children are presented with a feelings thermometer and asked to indicate how they are feeling from 0 to 10 (with 0 representing feeling happy/fulfilled/relaxed and 10 representing highest anxiety/distress ever felt).

#### Acceptability

Acceptability will be assessed through a series of 5-point Likert scale questions that are specifically designed for this intervention and based on our previous work [53]. Items such as “The workshop helped me feel better” are rated from strongly agree to strongly disagree. A series of open-ended questions regarding preferred and dispreferred components of the intervention, such as “what do you like about the workshop?” and “what did you not like?” are also asked to assess acceptability. Open-ended questions are also able to provide information on any unintended outcomes.

### Data Management

Hard copy data are collected and reviewed for any missing or inaccurate data. Data are then entered by a trained researcher. Data entry will be verified by a second researcher and all values inspected to ensure data quality. Hard copy data are securely stored in a locked filing cabinet. Unique identifier codes are stored separately to the data and accessible only to the project leads. Electronic data are securely stored on password-protected University cloud system. All data management and analyses are undertaken by the research team and are independent of the funding body. Adverse events will be identified by the research team through review of pre-post data upon collection. The research team will have access to the final deidentified dataset. Due to the small and short-term pilot nature of the study, no independent auditing systems are in place.

### Planned Data Analysis

Acceptability will be determined based on the acceptability Likert scale results as well as the open-ended survey feedback. Open-ended questions will be analyzed via content analysis, and Likert scale acceptability items will be descriptively reported. Feasibility will be assessed based on the completion of the program by individual participants, that is, the ability to recruit into the study and the proportion of participants completing the full program, with feasibility determined by recruiting the minimal sample size and an 80% completion/retention rate [54,55]. Utility will be demonstrated through improvements in subjective distress, mental health symptoms, or resilience between preintervention and postintervention. A paired-sample 2-sided *t* test will be performed for the outcome measures to identify any changes in the outcomes before and after the intervention. Baseline missing data will be removed pairwise, and analyses will be performed using the last observation carried forward.

### Ethical Considerations

This study has been approved by the University of New England’s Human Research Ethics Committee (HE23-080). Parental consent will be provided prior to children taking part in the study. The confidentiality and the nature of the study will be explained to the participants, and their assent will also be obtained prior to the commencement of the intervention. Study data will be deidentified, with participants assigned a unique code for linking their preintervention and postintervention data. Any potentially identifiable information provided in the open-ended responses will also be removed from any published materials to ensure privacy. Parents and children will be made aware that they are able to withdraw at any time without providing a reason and without consequence, and adherence will be recorded as the number of modules completed. Protocol amendments will be reported to the ethics committee and the trial registry. Catering during the intervention modules is provided, but no other compensation is provided to the participants.

## Results

Ethics approval was received in May 2023 (approval HE23-080). This study is being conducted over a 1-year period. As of March 2025, 28 children have been enrolled in the study. We expect results from this study to be published in late 2025. Results will be disseminated through conference presentations and workshops with practitioners, as well as through scientific publications.

## Discussion

### Overview

Intensifying climate change will exacerbate mental health symptoms and increase disorder prevalence, which will pose a significant strain for the mental health system [4]. Crucially, with escalating climate change, more individuals will be exposed to natural hazards and be more likely to face repeated exposures [10], with repeated exposure shown to further increase a range of mental health symptoms beyond single exposure alone [56]. Exposure to natural hazards also exacerbates climate change distress [11], further compounding the mental health sequalae of climate change. Accordingly, building resilience and adaptive capacity for future exposures is critical for protecting mental health. Interventions specifically addressing the mental health impacts of climate change and natural hazards are typically ad hoc and are not routinely or consistently delivered [57]. The evidence base also remains limited [31]. By building an evidence base for children, especially rural-dwelling children who are the most vulnerable to natural hazards, this study will contribute to informing mental health care by providing an evidence-informed intervention. Clinicians largely report feeling ill-equipped for treating eco-anxiety or climate-related distress in therapy [58], with practitioners in rural areas especially needing guidance on how to support mental health in relation to climate change [59]. Thus, this study will provide valuable guidance for clinicians. This brief group-based intervention to improve coping and resilience can be used to protect mental health in the future, which is critically needed to promote well-being as climate change escalates [60].

### Comparison With Prior Work

This study builds off the limited research evidence to date regarding supporting children and young people experiencing eco-anxiety, especially following exposure to a natural hazard. This study extends prior work by drawing on the preliminary and available evidence to date for promoting mental health and managing climate change–related distress. For example, this study brings together previously identified or suggested components to support young people, such as fostering meaning-making coping [38] and empowering children [22] such as through encouraging them to take action [31], balanced with also providing a safe and supportive space to explore and express negative emotional states [39]. Further, this study integrates aspects of ecotherapy and fostering a relationship with nature [31,40]. Although these components have previously been suggested as means through which individuals might be supported, the evaluation of any eco-anxiety intervention or treatment component has been minimal [3,31].

Current evidence for the treatment of eco-anxiety includes a qualitative evaluation of a dream-discussion–based group-processing program conducted with 7 adult climate activists [61]. Another evaluation study surveyed past participants of Carbon Conversations—a climate change education program of small, facilitated groups who meet 6 times to discuss topics related to climate change and allows for the discussion of emotional responses to climate change [62]. The results of this evaluation showed that 50% of those who responded to the survey rated the program as helpful for facing their worries about climate change; however, no preassessment/postassessment was undertaken, limiting the understanding of the effectiveness of the group in reducing any distress or mental health outcomes. Participants were also adults and predominantly older than 30 years. To date, the examination of eco-anxiety following exposure to natural hazards also remains minimal, and there are no treatments evaluated for this. Therefore, this study expands upon existing work by taking the proposed treatment components from prior work and testing an intervention specifically designed for children and adolescents.

### Limitations

Due to the nature of this pilot study, the sample size is small. This study is also not a randomized controlled trial, and random assignment to groups was not possible. Replication of results in randomized controlled trials with larger samples would be warranted after refinement of the intervention from the pilot. Although the current design is appropriate to gain insight into the effects of the intervention, the research would be strengthened by having longer follow-up times to better assess if the intervention effects are sustained. Outcome measures are child self-report only, and diagnostic interviews or parent reports may also be useful to incorporate into future research designs.

### Conclusions

This study tests a novel group-based intervention for supporting rural children who have experienced a natural hazard in relation to eco-anxiety. The impacts of climate change on mental health are rapidly gaining attention [63], and eco-anxiety has been widely shown to be detrimental for mental health [64]. Exposure to natural hazards also amplifies climate distress [11]. Children and young people are among the most vulnerable for eco-anxiety [65,66], with those in rural areas also more prone to experiencing repeated exposures to natural hazards, making rural children and young people an especially vulnerable population. Yet, empirical studies on eco-anxiety in children remain relatively minimal, with most studies being descriptive only [22]. Although the deleterious impacts of climate change on mental health are widely recognized, mental health clinicians report feeling underprepared for dealing with climate change [58], and service delivery remains fragmented after natural hazards [57]. To date, the evidence base for interventions for treating eco-anxiety remains sparse [3,31] and even more so for young people and children. This study will provide preliminary evidence of a group-based treatment for children and adolescents experiencing eco-anxiety that can inform practitioners.

## Appendix

Multimedia Appendix 1 Peer review report from the Peregrine Centre, Australia.

## Abbreviations

DASS-21: Depression Anxiety Stress Scale-21 items
SPIRIT: Standard Protocol Items: Recommendations for Interventional Trials

## Data Availability

Deidentified data are available from the authors upon reasonable request.
