# Supplementary material for: Maintaining Resilience and Well-Being in the Era of Climate Change: Protocol of an Acceptability and Feasibility Pilot of the Bee Well Program for Treating Eco-Anxiety in Rural Children Exposed to Natural Hazards
Source: JMIR Res Protoc. 2025 Jul 18;14:e69005. doi: 10.2196/69005 (PMC12317292; doi:10.2196/69005)
Supplement: Multimedia Appendix 1 [file resprot_v14i1e69005_app1.pdf]

The Peregrine Centre Pty Ltd
SmartSpace. Enterprise 1
Innovation Campus
University of Wollongong
Squires Way. Wollongong. 2500
info@theperegrinecentre.com.au
www.theperegrinecentre.com.au
(02) 4258 3400
ABN: 58 651 838 201
ACN: 651 838 201

Dr Suzanne Cosh
Associate Professor of Clinical Psychology
School of Psychology
University of New England
Armidale. NSW. 2351

13th March, 2023

Dear A/Prof Cosh,
We are pleased to inform you that you have been awarded a Small Project Grant as part of our Rural Mental Health Partnership with NSW Health. Your project entitled "The natural Disaster Resilience Project" will receive **$41,666** in total.

What happens next?

1. A 30-minute kick-off meeting with yourself and your research team is required. Please use this link to book yourselves into my diary, or email my Senior Admin: Kate.Newnham@theperegrinecentre.com.au . Below you will find some feedback from the selection panel, we ask that you are able to speak to these points when we meet.
2. After the meeting, we will forward a draft contract, including a payment schedule. Please send this through your organisation and let me know if you need to make any changes.
3. After the contract is executed, please ask your organisation to issue the first invoice directly to me at Rebecca.Sng@theperegrinecentre.com.au . As we need to distribute the funds by the end of the financial year, we would appreciate an invoice by May 30th, 2023.

Congratulations on your successful application! I look forward to meeting with your team to hear more about this exciting project.

Warm Regards,

Dr Rebecca Sng
Director
The Peregrine Centre

SELECTION PANEL FEEDBACK

1. The panel felt this application was well aligned with the research priorities and presented the opportunity for some innovative work within a project plan that looks achievable.
2. It was felt that this was a well-researched area (eg: Black Saturday Bushfires in Victoria) and it would be good to see evidence of building on the existing literature with this innovation. One suggestion was the work of Dr Rob Gordon with the Red Cross and his ideas about "Overwhelm" and multiple-faceted personalities promoting resilience.
3. The panel wondered if one of the published Disaster Preparedness scales could be included in the suite of measures.

The Peregrine Centre acknowledges the Traditional Owners of the land where we live and work.
We pay respects to Australia's First Peoples, to their unique and diverse cultures, and to Elders past, present and future.
